# Low-energy, Mobile Grain Boundaries in Magnesium

**DOI:** 10.1038/srep21393

**Published:** 2016-02-19

**Authors:** Xiangli Liu, Jian Wang

**Affiliations:** 1 Shenzhen Key Laboratory of Advanced Materials, Department of Materials Science and Engineering, Shenzhen Graduate School, Harbin Institute of Technology, Shenzhen 518055, P. R. China; 2 Mechanical and Materials Engineering, University of Nebraska-Lincoln, Lincoln, NE 68588, USA

## Abstract

The strong basal texture that is commonly developed during the rolling of magnesium alloy and can even increase during annealing motivates atomic-level study of dislocation structures of both <0001> tilt and twist grain boundaries (GBs) in Magnesium. Both symmetrical tilt and twist GBs over the entire range of rotation angles θ between 0° and 60° are found to have an ordered atomic structure and can be described with grain boundary dislocation models. In particular, 30° tilt and twist GBs are corresponding to energy minima. The 30° tilt GB is characterized with an array of Shockley partial dislocations ***b***_p_:-***b***_p_ on every basal _p_lane and the 30° twist GB is characterized with a stacking faulted structure. More interesting, molecular dynamics simulations explored that both 30° tilt and twist GBs are highly mobile associated with collective glide of Shockley partial dislocations. This could be responsible for the formation of the strong basal texture and a significant number of 30° misorientation GBs in Mg alloy during grain growth.

Rolled magnesium alloy sheets typically exhibit a strong basal texture with the c-axis of the crystals parallel to the through thickness direction of the sheet, leading to significant anisotropy of plastic deformations due to the drastic difference in the activity of unidirectional deformation twinning. Hence, weakening basal texture of magnesium (Mg) alloys is admired in advancing applications of Mg alloys. Several different thermomechanical processing techniques such as cross rolling^1^, asymmetric rolling^2^, twin roll casting^3^, and equal channel angular extrusion^4^ as well as alloy development^5^,^6^,^7^ have been applied as strategies to weaken the basal texture. For some applications, thermo-mechanical processing followed by various annealing schedules has been proven to obtain a recrystallization texture that replaces the deformation basal texture, as well as to relieve residual stresses. However, it has been noticed that the basal texture is retained^8^ or slightly weaken^9^ or even increased^10^,^11^ after recovery and recrystallization during annealing in Mg alloys. A considerable amount of research has been devoted to understand the basal texture development during grain growth of Mg alloys^7^,^8^,^9^,^10^,^11^,^12^,^13^.

It is well known that grain boundary energies are anisotropic and that the relative energies are influential in determining the polycrystalline structure. Results from experiments and simulations suggest an inverse relationship between the relative energy of a grain boundary and its total area in the polycrystal^14^,^15^,^16^,^17^,^18^. Lower-energy boundaries have, on average, larger areas than higher-energy boundaries. During annealing, growth of a grain is related to the mobility and the excess energy of its grain boundaries (GBs), as well as whether the grain is energetically advantageous to eliminate neighboring high energy grains and/or consume high energy GBs. Thus, low-energy GBs will be favorably developed during annealing, especially if they are highly mobile. From thermodynamical viewpoint, formation and evolution of the basal texture in Mg alloys are related to characters and properties of grain boundaries (GBs) that are corresponding to the crystallography of the basal texture wherein grains share the basal axis <0001>. The simplest and representative GBs are <0001> tilt GBs and (0001) twist GBs. Therefore, the main objective of this study is to characterize structures and migration mechanisms of both <0001> symmetrical tilt and twist grain boundaries at atomic level. Such studies may provide insightful knowledge into understanding formation and evolution of the basal texture in Mg alloys.

## Results

**Figure 1:**
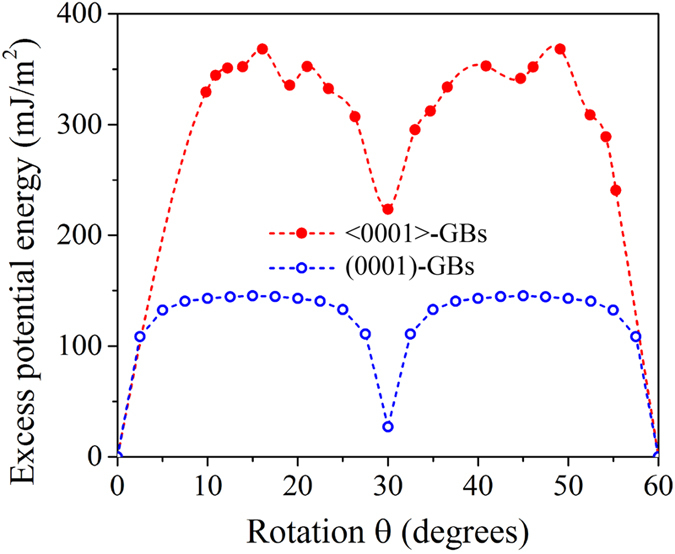
Excess potential energies of symmetrical tilt and twist grain boundaries, <0001>-GBs and (0001)-GBs, with respect to rotation angle *θ*.

**Figure 2:**
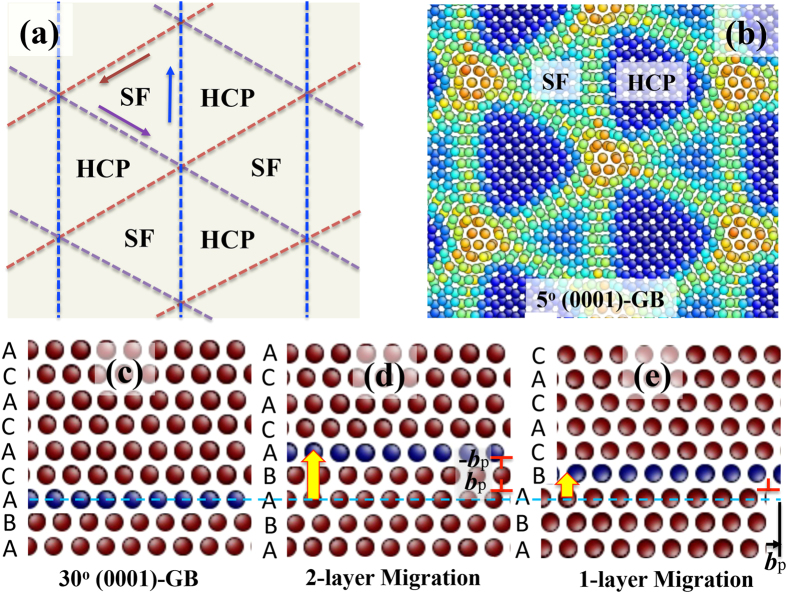
(**a**) Schematic of misfit dislocation networks on (0001)-GBs. (**b**) Atomic structure of the 5° (0001)-GB, that shows three sets of Shockley partial dislocations, normal HCP structures and stacking faulted structures (SF). The horizontal axis is along 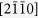, and the vertical axis is along 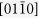. The arrows indicate Burgers vectors ***b***_p_ of three Shockley partial dislocations and the dashed lines show misfit dislocation lines. Atoms in (**b**) are colored according to their excess energy. (**c**) Atomic structure of 30° (0001)-GB, showing the stacking of hexagonal close compact planes AB**A**CACACA, where **A** is the shared boundary plane. The horizontal axis is along 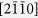, and the vertical axis is along [0001]. Two migration mechanisms: (**d**) 2-layer migration via the glide of a pair of Shockley partial dislocation dipole (***b***_p_:-***b***_p_) and (**e**) single layer migration via the glide of a single Shockley partial dislocation***b***_p_. The symbol “⊥” represents Shockley partial dislocation. The blue dashed line indicates the position of the initial faulted plane.

Using molecular dynamics method, we calculated the excess potential energy of <0001> symmetrical tilt GBs (referred to as <0001>-GBs) and (0001) symmetrical twist GBs (referred to as (0001)-GBs) in Mg as a function of rotation angle 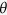, as shown in Fig. 1 (Approaches of assembling gain boundaries are described in Figure S1 in Supplementary). The two zero excess energy structures at the rotation angles 0° and 60° correspond to a perfect crystal. Between 0° and 60°, there is only one minimum energy GB at the rotation angle 30°. This is consistent with Electron Back Scatter Diffraction (EBSD) observations that a significant number of boundaries with 30° misorientation about the <0001> direction is characterized in Mg alloy AZ31B during grain growth^11^; and also satisfies the energetic criterion: low energy GBs favorably grow during annealing. The following issue is to test whether these low energy GBs are highly mobile.

symmetrical twist GB is semi-coherent and contains three sets of Shockley partial dislocations, as schematically shown in Fig. 2a. These dislocations have Burgers vectors of 1/3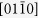, 1/3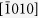, and 1/3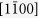 and separate the interface into two types of coherent structures: normal hexagonal close packed (HCP) structure (…ABABAB…) and stacking faulted (SF) structure (…ABAB**A**CAC…). A, B, and C represent three hexagonal close packed atomic planes with respect to (111) plane stacking in a face centered cubic (FCC) structure^19^,^20^. Due to the greater excess energy of the faulted structure than the normal HCP structure, misfit dislocation lines curve towards the SF regions associated with reducing the area of the faulted structure, decreasing interface excess energy as shown in Fig. 2b 19. With increasing the rotation angle from 0° to 30° or decreasing the rotation angle from 60° to 30°, the density of misfit dislocations increases. In other words, the spacing between misfit dislocations decreases in association with the increase of the number of misfit dislocation lines, as discussed in FCC-FCC (111) interfaces^21^,^22^,^23^. The 30° (0001)-GB is a stacking faulted coherent interface with the stacking …ABAB**A**CACA…, where the atomic plane **A** is shared by the two crystals (Fig. 2c). The migration of the 30° (0001)-GB can be accomplished through the glide of Shockley partial dislocations with two possible mechanisms. Figure 2d shows a two-layer migration mechanism via the glide of a pair of Shockley partial dipole on two neighboring {0001} planes. Associated with the GB migration through the 2-layer mechanism, there is no macro-scale strain because of the net zero Burgers vector^24^. Figure 2e shows a single layer migration mechanism via successive gliding of single Shockley partial dislocation on every {0001} plane, resulting in a macro-scale shear strain. Such Shockley partial dislocations can be generated by either the interaction of non-basal dislocations with GBs or the nucleation of partial dislocation at GBs^25^,^26^,^27^. Thus, the 30° (0001) GB is thermodynamically preferred during grain growth because of the low excess energy and high mobility associated with the glide of Shockley partial dislocations^28^.

**Figure 3:**
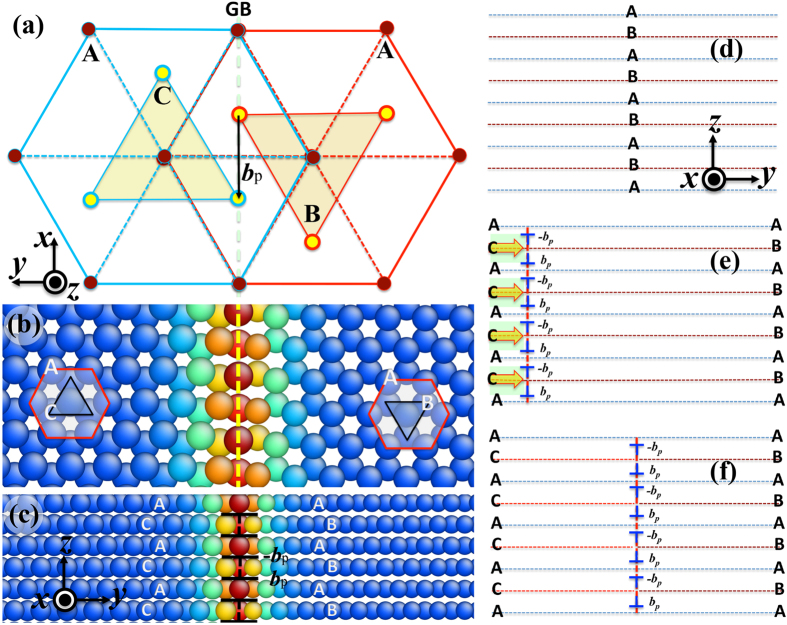
(**a**) Schematic of the 30° <0001>-GB, showing the left crystal with the stacks …ACAC… and the right crystal with the stacks …ABAB…. The grain boundary plane is {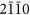}. The displacement for the transition from B to C is indicated by the black arrow and is equal to a Shockley partial dislocation. (**b**) and (**c**) atomic structures of the 30° <0001>-GB in two different views. (**d**) to (**f**) Schematics of creating a 30° <0001>-GB from a single crystal via the gliding of Shockley partial dislocations. A, B, and C planes can be referred to be (111) plane stacking with respect to face centered cubic structure. The x-axis is along 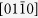, the y-axis is along 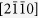, and the z-axis is along [0001]. Atoms in (**b**) and (**c**) are colored according to their excess energy.

<0001> symmetrical tilt GBs can be described with grain boundary dislocations as a tilt wall (atomic structures of several <0001>-GBs are shown in Figure S2 in Supplementary)^29^,^30^. The atomic structure of the lowest energy 30° <0001>-GB is shown in Fig. 3a,b. The right crystal has the stacks …ABABAB… and the left crystal has the stacks …ACACAC…. The migration of the GB towards the right involves the transformation of atomic planes B into atomic planes C. This can be accomplished with the glide of Shockley partial dipole 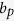 above and 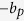 under the atomic plane B^31^,^32^. Therefore, the 30° <0001>-GB can be represented as an array of Shockley partial dislocations on every {0001} plane with a repeatable sequence 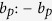, as shown in Fig. 3c; and can be created by gliding a set of partial dislocation dipoles in a single crystal (Fig. 3d–f)^24^. Accompanying with the glide of one pair of partial dislocation dipole on the neighboring {0001} planes, one atomic plane B shifts into plane C. Such gliding of a pair dislocation dipole will not generate strains due to the net zero Burgers vector. This is similar to Σ3{112} incoherent twin boundary in face centered cubic structure where three Shockley partial dislocations collectively glide on three (111) planes with the net zero Burgers vector^24^,^28^,^31^,^32^.

**Figure 4:**
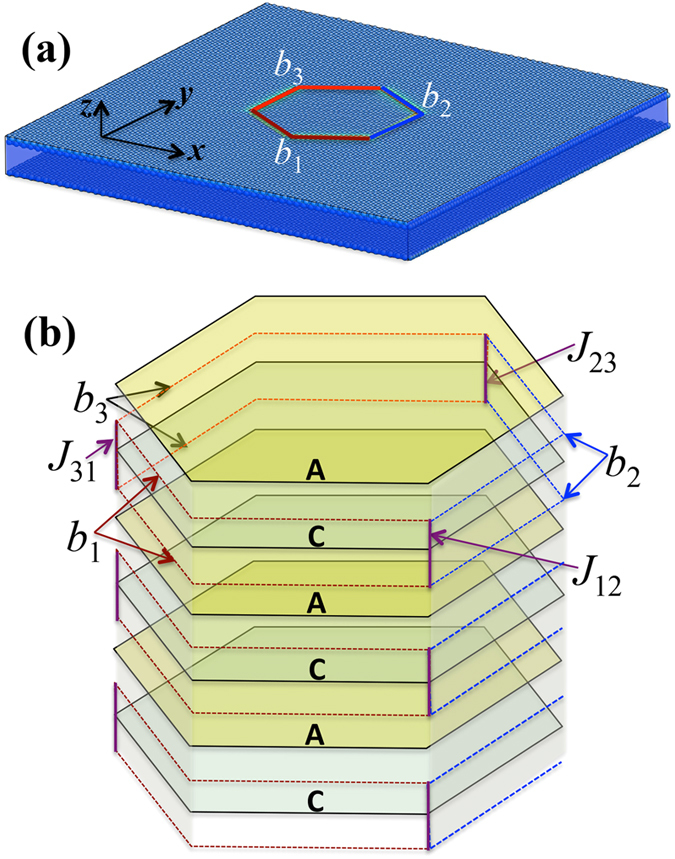
(**a**) A bi-crystal model to demonstrate migration of 30° <0001>-GB via molecular dynamics simulation. (**b**) Dislocation structures of the grain boundaries. Three dislocation loops (brown, red and blue dashed lines) with Burgers vectors *b*_1_, *b*_2_, and *b*_3_ present in the GBs by every two atomic planes and three jogs (purple straight segments), *J*_31_, *J*_23_, and *J*_12_ formed. The x-axis is along 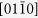, the y-axis is along 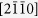, and the x-axis is along [0001].

Owing to high mobility of Shockley partial dislocation on (0001), the 30° <0001>-GB could be highly mobile corresponding to its dislocation structure. However, mechanical loading may not facilitate such motion because the net Peach-Kohler force acting on the partial dipole is equal to zero^33^,^34^. A generalized force, such as an elastic energy difference between the neighboring grains^18^, defect density difference in the neighboring grain^35^, or decreasing the curvature and/or area of grain boundaries^36^, can drive the migration of GBs. To test the mobility of the 30° misorientation GBs, we create a bi-crystal structure: a hexagonal pore grain is embedded in the matrix according to the misorientation of the 30° <0001>-GB (Fig. 4a). The hexagonal pore grain has the side length of 6 nm. Six GBs have boundary planes 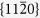. According to the dislocation structure of the 30° <0001>-GB, grain boundaries in the bi-crystal can be characterized with three Shockley partial dislocation loops, 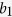,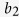 and 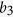, and their junctions, 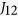,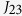 and 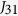, as shown in Fig. 4b. The bi-crystal structure is relaxed under zero applied stresses at room temperature of 300 K. The details can be found in Figure S3 in Supplementary.

**Figure 5:**
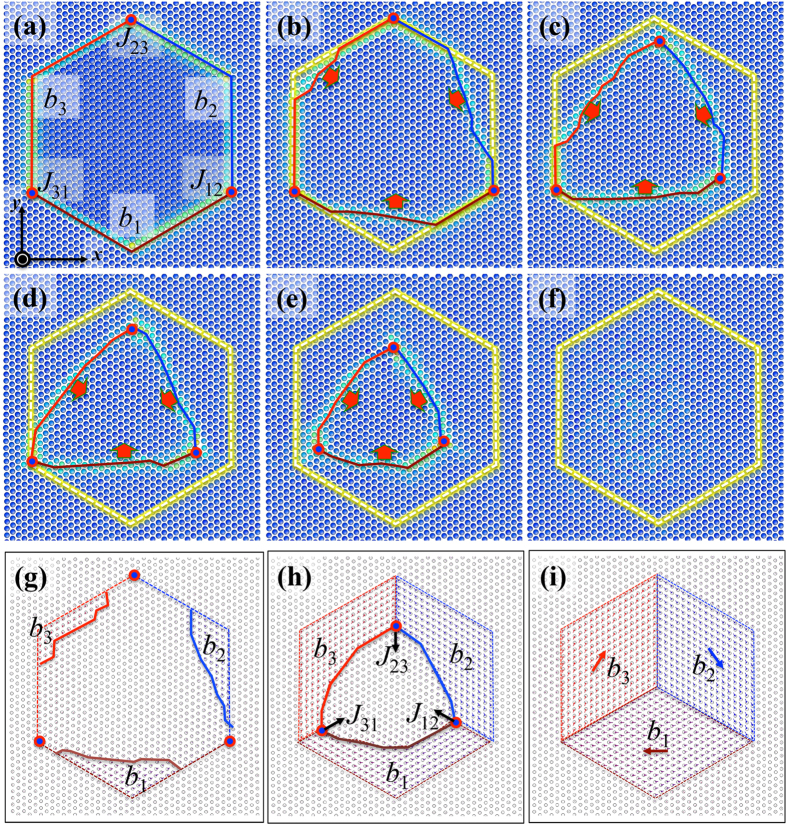
(**a–f**) Plan-view of the bi-crystal structure, six snapshots shows the migration of GBs via collective glide of Shockley partial dislocations and their jogs. Yellow hexagon outlines the initial GBs. Three bold lines colored in the red, the brown, and the blue, represent the three Shockley partial dislocations with Burgers vectors *b*_1_, *b*_2_, and *b*_3_, respectively. Three jogs, *J*_31_, *J*_23_, and *J*_12_ are marked in the red circles. (**g–i**) Disregistry plots of three atomic structures in (**b**), (**e**), and (**f**) respectively, showing shear displacements after the glide of Shockley partial dislocations. The x-axis is along 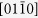, the y-axis is along 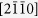, and the z-axis is along [0001]. Atoms are colored according to their excess energy.

MD simulations demonstrated the migration of the GBs (also see Movie I in Supplementary) and revealed migration mechanisms of the GBs — the collective glide of Shockley partial dislocations — as evidenced by six snapshots in Fig. 5. To identify Burgers vectors of these Shockley partial dislocations that present in the GBs, we performed disregistry analysis across {0001} planes (Fig. 5g–i)). The results indicate that the six GBs can be represented as three sets of repeatable dislocation loops with Burgers vectors *b*_1_, *b*_2_, and *b*_3_ on every two atomic planes and three junctions. The junction formed by two dislocation loops is a jog with Burgers vector of 1/3<
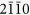
> (e.g., the junction formed by the dislocation loops *b*_3_ and *b*_1_ is referred to as the jog *J*_31_. Its Burgers vector is equal to *b*_3_−*b*_1_). These jogs are mobile because their Burgers vectors are along the compact direction on {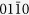} planes. Thus, the hexagonal pore grain is surrounded by three mobile jogs, *J*_12_ *J*_23_, and *J*_31_, and three Shockley partial dislocation loops *b*_1_, *b*_2_ and *b*_3_, as shown in Fig. 4b. During MD relaxation, it is noticed that the curved segment of each dislocation loop commences to move, driven by reducing the curvature of the GBs. The two partials on the adjacent glide planes glide together because they attract each other due to the same Burgers vector while the opposite line sense as shown in Fig. 4b (or they form a pair of dislocation dipole as shown in Fig. 3c). Reducing the area of GBs in the bi-crystal motivates the continuous glide of partial dislocations and jogs. The three jogs and dislocations move and finally meet together. The summation of Burgers vectors of these dislocations is equal to zero. Thus, the hexagonal pore grain finally transforms into the same orientation as the surrounding grain, forming a single crystal (Fig. 5f).

## Discussion

The strong basal texture is commonly developed during the rolling of Mg alloy (AZ31) and can even increase during annealing. Using Electron Back Scatter Diffraction (EBSD) analysis, a significant number of boundaries with 30° misorientation about the <0001> direction is characterized in Mg alloy AZ31B during grain growth. From thermodynamical viewpoint, texture evolution is controlled by boundary anisotropy in energy and mobility. This motivates atomic-level study of dislocation structures and migration mechanisms of both <0001>-GBs and (0001)-GBs in Magnesium.

Atomistic simulations so far revealed that both 30° <0001>-GB and (0001)-GB are energetically favored during grain growth due to the low excess formation energy, compared with other <0001>-GBs and (0001)-GBs, and even compared with <
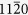
> and <
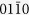
> tilt grain boundaries (Figure S5 in Supplementary)^29^,^30^. The 30° (0001)-GB is characterized to be a stacking faulted structure and can migrate through the glide of Shockley partial dislocation. Atomic structure of the 30° <0001>-GB is characterized to contain a repeatable sequence of Shockley partial dislocation dipoles …
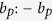
…. Such dislocation patterns imply that 30° <0001>-GBs are highly mobile due to the easy glide of Shockley partial dislocation on (0001) plane. Using molecular dynamics simulation in a bi-crystal, we demonstrated the easy migration of 30° <0001>-GBs through collective glide of Shockley partial dislocation dipoles and their junctions. It is noticed that the migration of these GBs does not result in macro-scale shear strains because of the net zero Burgers vector. The similar phenomena and mechanisms have been observed and demonstrated in nanotwinned Cu and Ag, and phase transformation in InAs nanowirs using *in situ* microscopes and atomistic simulations^31^,^32^,^33^, wherein three sets of Shockley partial dislocations with the net zero Burgers vector collectively glide, resulting in zero-strain twinning and detwinning. Migration of such GBs does not require mechanical loading due to the net zero PK force, but can be driven by decreasing grain boundary curvature, or by a generalized force, such as an elastic energy difference and/or the defect density difference between the neighboring grains, which is generally resulted after several mechanical deformation.

However, the conclusion drawn so far is based on molecular statics/dynamics calculations for ideal 30° <0001>-GBs and 30° (0001)-GBs in Mg, regardless of temperature, impurity, and additional grain boundary defects including vacancy, interstitial, and disconnections etc. In real materials, such defects are often present within GBs. In addition, materials were synthesized or treated at different temperatures and/or during different mechanical processes, internal stresses during these processes will be built and may change the structure of GBs. Even for most thermally stable twin boundaries in fcc metals, GBs associated with twin orientation relationships can contain different facets that vary with temperature and impurity, which corresponds to the faceting-roughening transformation mechanisms^37^. Corresponding to the dislocation structure of ideal 30° <0001>-GBs and 30° (0001)-GBs in Mg, high temperature will reduce the Pierles stress of Shockley partial dislocations, facilitating the motion of Shockley partial dislocations. As a consequence, GBs deviating from ideal 30° <0001>-GBs will be thermodynamically transformed to faceted GBs containing nearly ideal 30° <0001>-GBs, which is driven by reducing GBs energy. Corresponding to the low energy and high mobility of both 30° <0001>-GBs and 30° (0001)-GBs, these boundaries may dominate the grain growth phenomenon, leading to the growth of basally oriented grains, which results in the strengthening of the texture intensity.

## Methods

Atomistic models including topological models and molecular static/dynamic simulations are employed to characterize structures and properties of <0001> tilt and (0001) twist boundaries in Mg. Here we studied symmetric tilt and twist grain boundaries that are the simplest of <0001> tilt and twist GBs and only required two parameters (the tilt/twist axis and tilt/twist angle 2*θ*) to describe their crystallographic relations^29^,^30^. For simplicity of the following description and discussion, <0001> symmetrical tilt grain boundaries are referred to as <0001>-GBs and <0001> symmetrical twist grain boundaries are referred to as (0001)-GBs. We employ molecular dynamics (MD) simulations to characterize atomic structures of <0001>-GBs and (0001)-GBs over the entire range of the rotation/twist angle *θ*. In the fixed coordinate system (Fig. S1a), the *x*-axis lies along 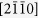, the *y*-axis lies along 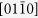, and the *z*-axis lies along [0001]. The two crystals originally adopt the same local coordinate system as the fixed coordinate system. For <0001>-GBs, the *z*-axis is the tilt axis and the *z*-*x* plane is the GB plane. The two crystals rotate an angle 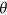 clockwise and counterclockwise about the *z*-axis, respectively (Figure S1b). The corresponding MD simulation cell containing a single GB on the *z*-*x* plane is illustrated in Figure S1c. Symmetrical twist grain boundaries are assembled using the same operation with the boundary plane on the *x*-*y* plane. The two crystals twist around the *z*-axis. In MD simulations, the simulation model contains two parts — a moveable region inside the simulation cell and a semi-rigid region that surrounds the movable region. The semi-rigid region acts as a flexible boundary to mimic the bulk response during MD relaxation^38^. For <0001>-GBs, periodic boundary conditions are applied in both the *x* and *z* directions. The *x* dimension varies with respect to the tilt angle 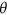 such that periodic boundary conditions are satisfied. The height of the two crystals in the *y* is 8.0 nm and the thickness of semi-rigid regions in the *y* is 1.2 nm, which is two times the cutoff of the potential used. The dimension in the *z* direction is ~2.6 nm. For (0001)-GBs, we adopted a cylindrical bi-crystal with the longitude axis along the z-axis and the radius of 10 nm. The height of the two crystals in the *z-*axis is 10.0 nm and the thickness of semi-rigid regions is 1.2 nm in the radial direction and in the *z-*axis (Fig. S1d).

The bi-crystal models are relaxed at 0 K by quenching molecular dynamics using an embedded atom method (EAM) potential for Mg^39^. This EAM potential reproduces well many experimentally measured properties and predictions of defect formation energies using first principle density function theory calculations. The predictor-corrector algorithm developed by Gear was used in our simulations with a temp step of 0.002 ps^40^. During MD relaxation, the two crystals are allowed to translate in the *x*-*y*-*z* directions^41^. Energy minimization ends when the maximum force acting on any atom in the system does not exceed 5 pN.

## Additional Information

**How to cite this article**: Liu, X. and Wang, J. Low-energy, Mobile Grain Boundaries in Magnesium. *Sci. Rep.* **6**, 21393; doi: 10.1038/srep21393 (2016).

## Supplementary Material

Supplementary Information

Supplementary video
