# Supplementary material for: Low-energy, Mobile Grain Boundaries in Magnesium
Source: Sci Rep. 2016 Feb 19;6:21393. doi: 10.1038/srep21393 (PMC4759561; doi:10.1038/srep21393)
Supplement: Supplementary Information [file srep21393-s1.pdf]

Supplementary Information

Low-energy, Mobile Grain Boundaries in Magnesium

Xiangli Liu[1], Jian Wang[2,*]

[1] Shenzhen Key Laboratory of Advanced Materials, Department of Materials Science and Engineering, Shenzhen Graduate School, Harbin Institute of Technology, Shenzhen 518055,P. R. China

[2] Department of Mechanical and Materials Engineering, University of Nebraska-Lincoln, Lincoln, NE 68588, USA

*Corresponding author: Dr. Jian Wang, Phone: +1 402-472-2375, E-mail: jianwang@unl.edu

Additional information includes

Figure S1. Schematics of assembling tilt and twist grain boundaries.

Figure S2. Atomic structure of <0001>-GBs at different tilt angles.

Figure S3. Simulation cell of a bi-crystal structure.

Figure S4. Comparison of boundary formation energies.

Movie I. Atomistic simulation shows migration of 30° <0001>-TBs.

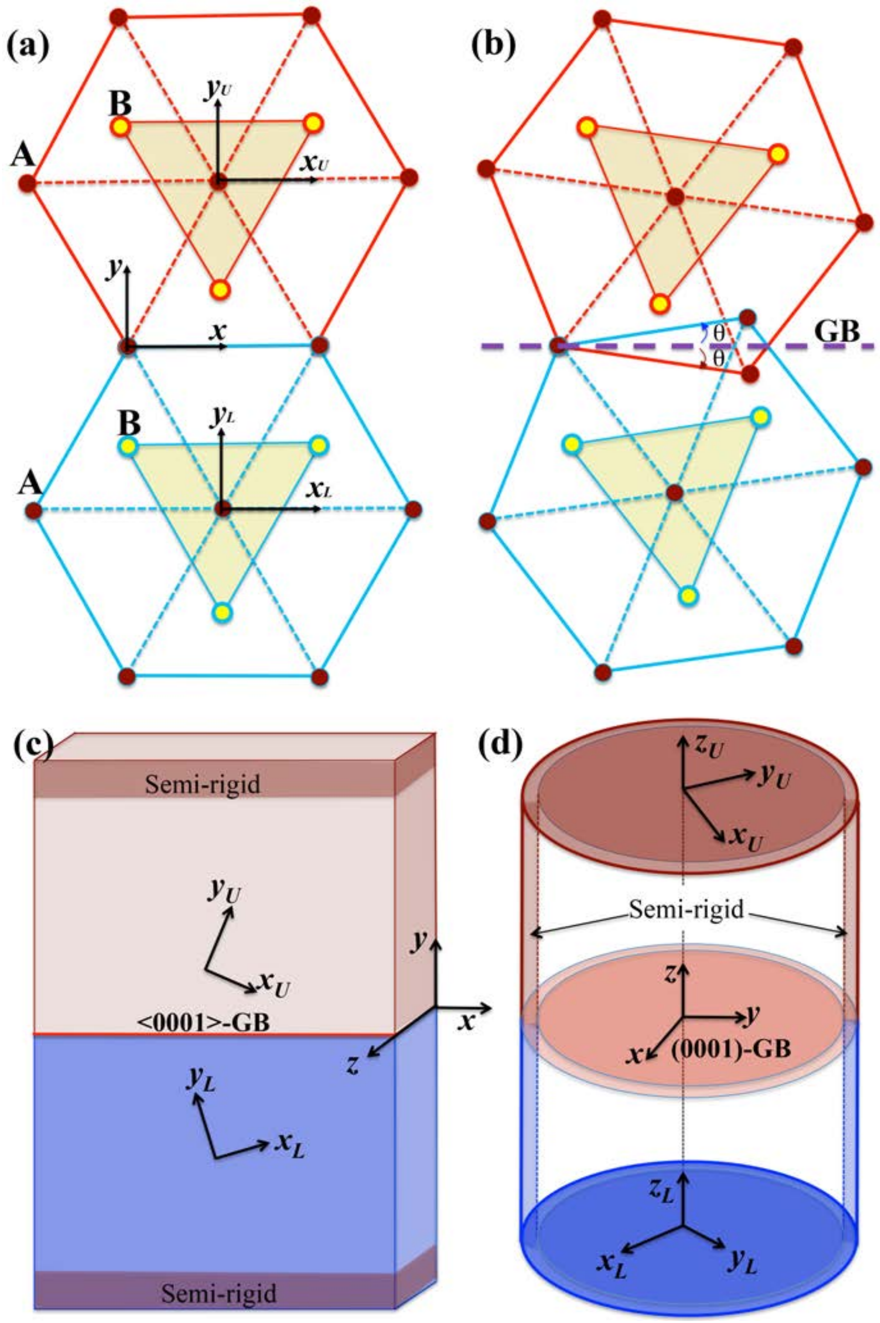

Figure S1. (a) and (b) schematics of assembling tilt and twist grain boundaries about the tilt and twist axis <0001>. (c) and (d) Molecular dynamics simulation cells of symmetrical tilt and twist grain boundaries. A and B represent adjacent atomic planes {0001}.

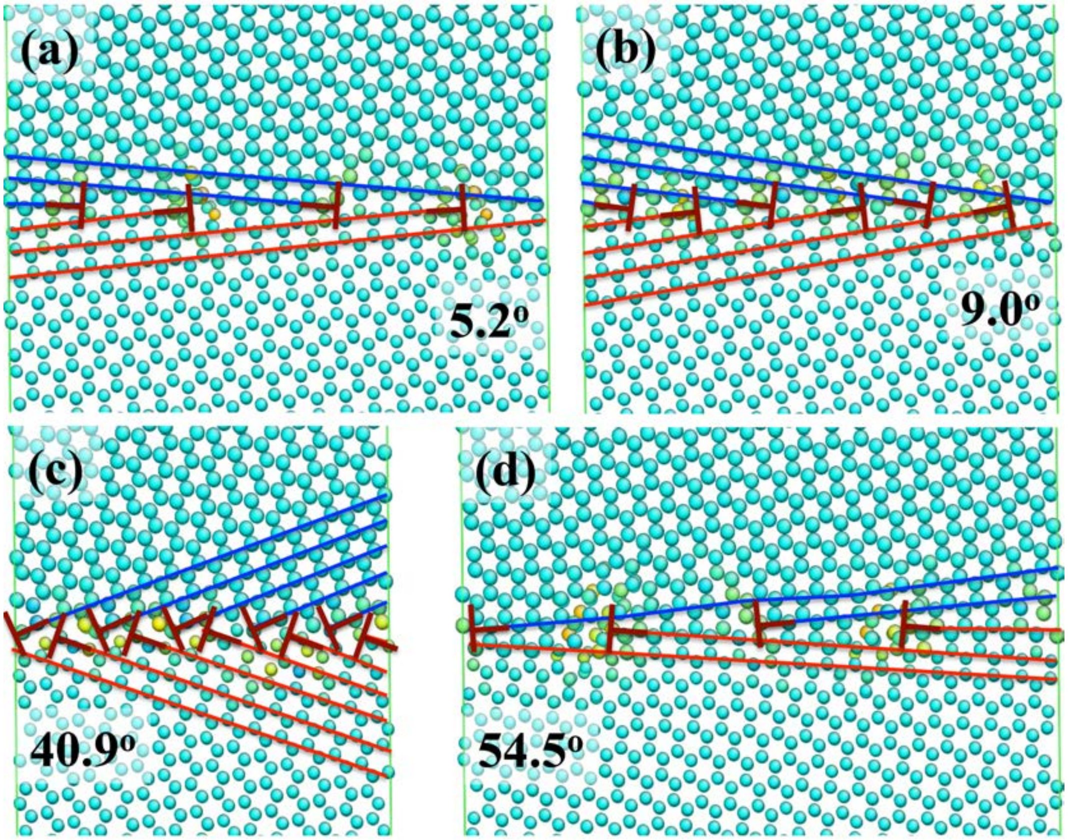

Figure S2. Atomic structure of symmetrical tilt <0001>-GB at different rotation angles, (a) 5.2^{o}, (b) 9.0^{o}, (c) 40.9^{o}, and (a) 54.5^{o}. The tilt axis is about <0001> pointing out of the paper. The blue and red lines indicate prismatic plane in the upper and lower crystals. The symbol "⊥" represents grain boundary dislocation with Burgers vector of $1/2 < 01\bar{1}0 >$. The calculated average separation between GBDs according to Frank's formula,[1] i.e., $\langle L \rangle = |\boldsymbol{b}_{GB}|/\sin(\varphi)$, are consistent with the measured separation from the relaxed MD models. Where φ is the tilt angle $\varphi = 2\theta$, $|\mathbf{b}_{GB}|$ is the magnitude of the Burgers vector of GBDs.[2,3]

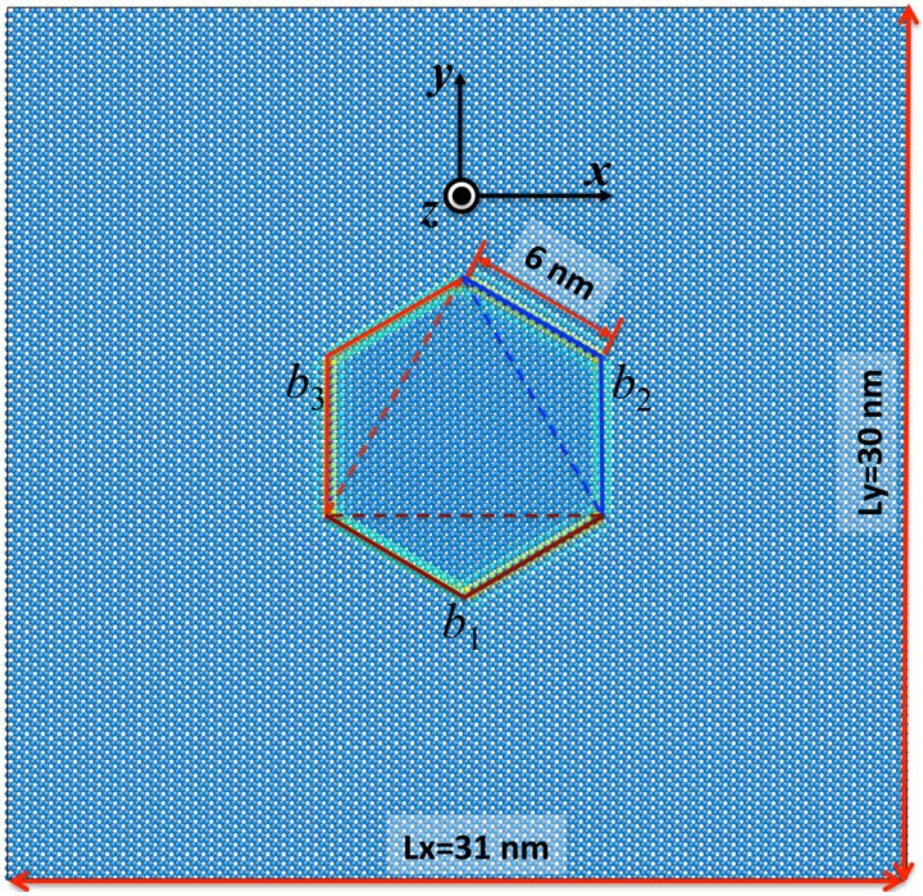

Figure S3. Simulation cell of the bi-crystal wherein a hexagonal pore grain embedded in the matrix has six 30° <0001>-GBs with grain boundary planes $\{01\bar{1}0\}$. The dimensions of the MD simulation cell are 31 nm in the *x*-direction ($[01\bar{1}0]$), 30 nm in the *y* direction (($[2\bar{1}\bar{1}0]$), and 3.2 nm in the *z* direction ([0001]). The hexagonal pore grain has the side length of 6 nm. Periodic boundaries are applied in the *x*-, *y*-, and *z*-axis. The bi-crystal structure is then relaxed under zero applied stresses at room temperature of 300 K. The three dashed lines indicate three $\{2\bar{1}\bar{1}0\}$ planes corresponding to the 30° <0001>-symmetrical tilt GBs. For the given misorientation, the content of interface dislocations does not change with the boundary planes. Thus, the six 30° <0001>-GBs with boundary plane $\{01\bar{1}0\}$ can be described with a repeatable sequence of Shockley partial dislocation dipoles $\ldots b_p\!: -b_p \ldots$, where p is equal to 1, 2, and 3.

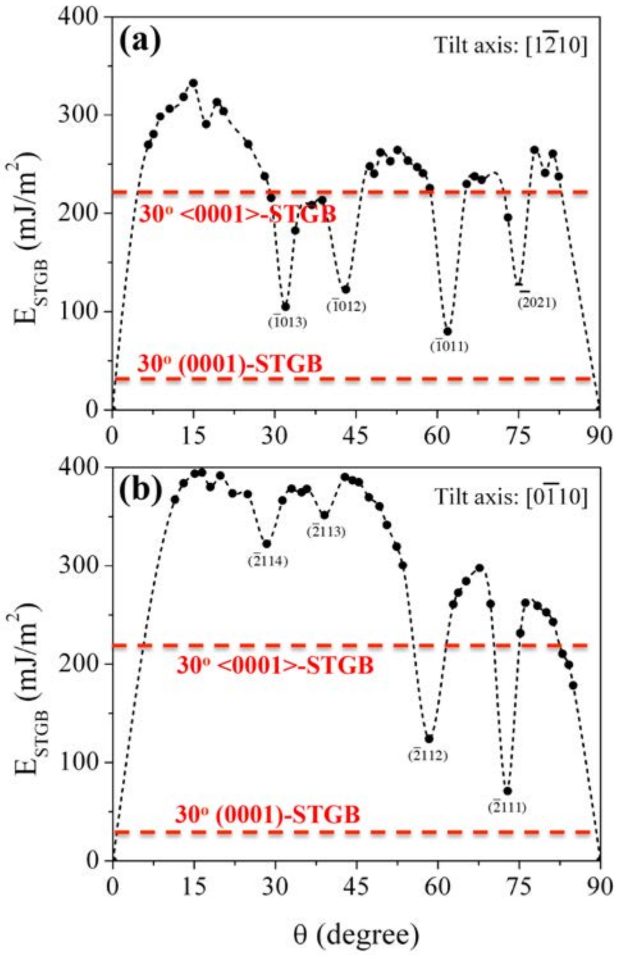

Figure S4. Comparison of excess formation energies of <1$\bar{2}$10> and <0$\bar{1}$10> symmetrical tilt grain boundaries in Mg.[2,3] 30° <0001>-symmetrical tilt GB and 30° (000)>-symmetrical twist GB have low excess formation energy than most of other tilt GBs, implying that the development of the basal texture is thermodynamically favored during grain growth.

References

1. Hirth, J. P. & Lothe, J. *Theory of dislocations*. Wiley. (1982).
2. Wang, J. & Beyerlein, I. J. Atomic structures of symmetric tilt grain boundaries in hexagonal close packed (hcp) crystals. *Model. Simul. Mater. Sci. Eng*. **20**(2), 024002 (2012).
3. Wang, J. & Beyerlein, I. J. Atomic structures of <0-110> symmetric tilt grain boundaries in hexagonal close-packed (hcp) crystals. *Metal. Mater. Trans. Part A* **43**(10), 3556-3569 (2012).
